# Chloroplast protrusions in leaves of *R* *anunculus glacialis* L. respond significantly to different ambient conditions, but are not related to temperature stress

**DOI:** 10.1111/pce.12483

**Published:** 2015-01-23

**Authors:** TIM MOSER, ANDREAS HOLZINGER, OTHMAR BUCHNER

**Affiliations:** ^1^ Institute of BotanyUniversity of InnsbruckInnsbruckA – 6020Austria

**Keywords:** climate conditions, stroma, stromules, thylakoid, ultrastructure

## Abstract

The occurrence of chloroplast protrusions (CPs) in leaves of *R* *anunculus glacialis* L. in response to different environmental conditions was assessed. CPs occur highly dynamically. They do not contain thylakoids and their physiological function is still largely unknown. Controlled *in situ* sampling showed that CP formation follows a pronounced diurnal rhythm. Between 2 and 27 °C the relative proportion of chloroplasts with CPs (rCP) showed a significant positive correlation to leaf temperature (TL; 0.793, *P* < 0.01), while irradiation intensity had a minor effect on rCP. *In situ* shading and controlled laboratory experiments confirmed the significant influence of TL. Under moderate irradiation intensity, an increase of TL up to 25 °C significantly promoted CP formation, while a further increase to 37 °C led to a decrease. Furthermore, rCP values were lower in darkness and under high irradiation intensity. Gas treatment at 2000 ppm CO_2_/2% O_2_ led to a significant decrease of rCP, suggesting a possible involvement of photorespiration in CP formation. Our findings demonstrate that in *R* *. glacialis*, CPs are neither a rare phenomenon nor a result of heat or light stress; on the contrary, they seem to be most abundant under moderate temperature and non‐stress irradiation conditions.

## Abstract 2

Chloroplast protrusions (CPs) are stroma‐filled areas not containing any thylakoids. They are formed dynamically and the physiological function is still largely unknown. We conducted field and laboratory experiments on the nival plant species Ranunculus glacialisL. and demonstrate that CP formation follows a pronounced diurnal rhythm. CPs are neither a result of heat or light stress but seem to be most abundant under moderate temperature and non‐stress irradiation conditions.

**Abbreviations**
CP: chloroplast protrusion, respectively, a chloroplast showing at least one protrusion
F_v_/F_m_: potential efficiency of photosystem II
GA: glutaraldehyde
PCC: Pearson product–moment correlation coefficient
P_n_: net CO_2_ assimilation rate (*μ*mol CO_2_·m^−2^·s^−1^)
PPFD: photosynthetically active photon flux density (*μ*mol photons·m^−2^·s^−1^)
PPFD10: PPFD intensity averaged over 10 min
RuBP: ribulose‐1,5‐bisphosphate
TEM: transmission electron microscopy
TL10: leaf temperature averaged over 10 min

## Introduction

Even before the application of the first transmission electron microscopes (TEM) for examining biological samples, light‐microscopists described protuberances of the chloroplast envelope in various bryophytes and the green alga *Bryopsis* (Senn 1908) as well as in higher plants (e.g. *Colchicum bornmülleri*; Heitz 1937). They were characterized as pseudopodia of the chloroplast envelope, then referred to as ‘Peristromium’, and some investigators assumed that they might facilitate amoeboid movement of the chloroplasts (Senn 1908).

Advances in light and electron microscopy led to the discovery and rediscovery of amoeboid plastids in a range of plant species and plant organs over the subsequent decades (see Gray *et al*. 2001; Kwok & Hanson 2004; Hanson & Sattarzadeh 2008, 2011).

Because of the variability of these protuberances, it was suggested that they could be divided into two types. Wide protuberances and bulges were more often observed by electron microscopy, and thinner, longer extensions were observed by phase‐contrast microscopy (Thomson 1974).

Permanent thylakoid‐free broad chloroplast protuberances, termed ‘proliferations’, were depicted in TEM images of *Ranunculus glacialis* (Lütz & Moser 1977; Lütz 1987) and *Oxyria digyna* (Holzinger *et al*. 2007b; Lütz & Engel 2007). These proliferations were frequently observed near peroxisomes and mitochondria, suggesting that photorespiration might be involved as a driving factor in their formation (Lütz & Moser 1977; Lütz 1987).

Visualization of stroma‐located transit peptides using green fluorescent protein (GFP) led to impressive images (Köhler *et al*. 1997) and videos (Köhler & Hanson 2000; Gunning 2009) of chloroplasts forming tubular extensions in living cells of petunia and tobacco plants. The extensions were termed ‘stromules’ and defined as stroma‐filled tubular extensions that appear to be less than 0.8 *μ*m in diameter (Köhler & Hanson 2000). The function of stromules is still under discussion, especially regarding the exchange of metabolites and proteins (Hanson & Sattarzadeh 2011, 2013; Schattat *et al*. 2012, 2014).

To distinguish broad beak‐like proliferations, later referred to as chloroplast protrusions (CPs), from these stromules, a ‘shape index’ was determined (Holzinger *et al*. 2007a). Whereas the ratio of a CP's length to half the width of its base was 0.8 ± 0.3, the same ratio for stromules was 7.0 ± 1.3 in *Arabidopsis thaliana* (Holzinger *et al*. 2007a).

While these two types of protuberances clearly have different shapes, both are highly dynamic, thylakoid‐free and stroma‐filled regions of the chloroplast and the supposition that their functional role is somewhat similar cannot be ruled out. However, the present study focused solely on CPs and not on stromules.

In Sitka spruce needles, CPs were observed after treatment with acid mist (Wulff *et al*. 1996). Paramanova *et al*. (2004) observed CPs in salt‐stressed leaves of *Mesembryanthemum crystallinum*, many of them containing degraded starch. A more recent study showed that salt stress may also lead to the formation of CPs in rice. Furthermore, it was shown that these salt‐induced CPs contain the enzyme rubisco, and in some cases, crystalline inclusions. The presence in the cytosol of membrane‐encased bodies containing both rubisco and crystalline inclusions suggests that CPs may play a role as a pathway for rubisco and crystallized storage proteins to be excluded from the chloroplast (Yamane *et al*. 2012). Salt‐stress treatment of soybean likewise resulted in an increase in the number of CPs, as well as rubisco‐containing bodies (RCB) and a significant decrease in overall rubisco content and photosynthetic activity (He *et al*. 2014). Ishida *et al*. (2014) conclude that RCB and other vesicles originating from stromules or CPs are involved in specific autophagic processes that play a role in nutrient recycling and chloroplast function maintenance.

The influence of irradiation intensity and temperature on the formation of CPs has been under investigation. Utilizing a temperature‐controlled chamber to decouple temperature and irradiation emitted by the microscope illumination, it was shown in Buchner *et al*. (2007a, 2007b) that temperature especially had a significant effect on CP abundance. This response to changes in temperature was observed in leaf sections of many high alpine and nival plant species. Additionally, it has been shown that various high alpine plants contain numerous CPs under fully natural conditions (Lütz & Engel 2007), although the conditions prevailing at the time of sampling were not monitored.

Recently, Machettira *et al*. (2012) demonstrated that chloroplast membrane morphology can be modulated by membrane proteins, and suggested that membrane protrusions might reflect a compensatory mechanism of membranes to maintain the ratio of proteins and lipids constant during the insertion of large amounts of membrane proteins.

Despite these findings related to the occurrence of CPs, both the physiological function and the factors promoting the formation of CPs are largely unknown and not understood. Therefore, for the first time, this study combined precise microclimate measurements, field work and laboratory techniques to further illuminate the natural conditions responsible for CP formation.

(1) *In situ* sampling at different times of the day followed by immediate chemical fixation was performed in order to determine if CP abundance in *R. glacialis* follows a natural diurnal rhythm. (2) Neutral density filters were used to manipulate irradiation intensity *in situ*, and full decoupling of temperature and irradiation was realized in a laboratory experiment where different temperature/irradiation combinations were applied to whole individuals. A response of CP abundance to variations in these factors may imply a direct connection to changes in photosynthetic and overall physiological activity. (3) Finally, exposure of whole individuals to high photosynthetically active photon flux density (PPFD), but at different CO_2_/O_2_ ratios should indicate possible connections between net photosynthetic assimilation rate (P_n_) or photorespiratory activity and CP formation.

In summary, this study was aimed at testing if the time of day affects CP, if the effect is caused primarily by temperature or by light, if CP formation is directly linked to photosynthesis and related pathways, and, finally, if it is related to temperature stress.

## Material and Methods

### Plant material and study site

*R. glacialis* L. is a small hemi‐cryptophytic species occurring in nutrient‐poor siliceous rocky habitats, in the upper alpine and nival zones of the European Alps as well as in arctic and subarctic regions. Growing 5–25 cm tall and covering up to 100 cm^2^, it forms white flowers (12–30 mm diameter) that turn crimson with senescence. The three‐lobed leaves are fleshy and dark green, and vary widely in shape. *R. glacialis* is one of the highest‐ascending seed plants in the European Alps.

The study site was located close to Timmelsjoch (2563 m a.s.l., Ötztal Alps, Tirol, Austria; N46° 53′ 42.245″, E11° 5′ 57.523″) in a depression of the mountain ridge where wind speeds are lower and snowmelt is delayed. All individuals used for the experiments grew in soil that remained humid during the summer months. For laboratory experiments, plants were dug out with intact roots (complete with surrounding soil), potted, and transferred to the ‘Alpine Garden Mt. Patscherkofel’ (1950 m a.s.l., Tirol, Austria) for further processing. The plants used in the temperature/irradiation experiment were transported directly to the Institute of Botany of the University of Innsbruck and were temporarily kept in a cold room (5 °C) and illuminated for 8 h a day at PPFD of 100 µmol photons·m^−2^·s^−1^.

### Microclimate

Prior to sampling, a microclimate station was installed *in situ*. Thirteen thermocouples (Type T, GG‐Ti‐28 and TT‐Ti‐40, Omega Engineering, Stamford, CT, USA), one soil temperature sensor (*R. glacialis* root area; 108 Temperature Probe, Campbell Scientific, Loughborough, UK) and one quantum sensor (40 cm above ground; QS, Delta‐T Devices, Cambridge, UK) were connected to a datalogger and multiplexer unit (CR1000 and AM25T, Campbell Scientific). Two thermocouples were used to measure ambient air temperature at 30 and 200 cm above the ground, and 11 temperature sensors were mounted on leaves of *R. glacialis* plants by means of small magnetic clips (described in detail by Buchner *et al*. 2013). Values were recorded once a minute from the beginning of August till the end of September 2012.

### Light microscopy assessment of relative CP numbers (rCP)

Semi‐thin sections (30 *μ*m) of glutaraldehyde (GA)‐fixed leaf samples stored in sodium cacodylate buffer (SC‐buffer) were sliced and analysed using differential interference contrast optics with an inverted microscope (Axiovert 200 M; Plan‐Apochromat 63 × 1.4 Na; Carl Zeiss, Jena, Germany). For each sample, 10 palisade parenchyma cells were photographed (Axiocam MCR 5, Carl Zeiss). Stacks of images showing the same cell were analysed at different focal planes (Adobe Photoshop CS2, Adobe Systems, Inc., San José, CA, USA). For each cell, 10 chloroplasts were thoroughly screened for CPs. The selected chloroplasts were slightly off the cell wall, not concealed by cell wall fragments or other structures, and close to each other. rCP was calculated for each cell screened (Eqn (1)).

(1) $$rCP\left[\%\right]=\frac{n_{\text{CP}}}{n}\cdot 100$$

Where, *n*_CP_ is the number of chloroplasts showing at least one CP, *n* is the number of chloroplasts inspected.

If not indicated otherwise, the rCP values of cells belonging to the same leaf sample were not averaged, but were treated as individual values.

### Effect of daytime: diurnal course of CP occurrence

Leaves of untreated healthy *R. glacialis* plants were GA‐fixed *in situ* during the course of 2 d. Samples were taken nine times on 11–12 August 2012 (10:20, 12:18, 14:20, 16:26, 18:28, 20:26, 21:30, 06:00, 08:15) and four times on 18 August 2012 (9:06, 12:42, 15:14, 18:56). At any sampling time, one leaf from each of the three different individuals was cut into 2 × 2 mm pieces and fixed using 2.5% GA in 50 mM, pH 7.0 SC‐buffer. After 1.5 h, the leaf pieces were rinsed with and subsequently stored in SC‐buffer. In total, 39 samples were collected throughout the two runs of the experiment. They were analysed under a light microscope, and the results were related to the respective microclimate data.

### Effects of leaf temperature and irradiation intensity on rCP

#### Effect of *in situ* shading on rCP

Under otherwise fully natural conditions, individual plants were either unshaded or shaded *in situ* with two or four neutral density filters (250 × 250 mm, NG 11, Schott, Mainz, Germany) that were placed 5 cm above the plants. The light absorbance of the filters was 78.1% for two and 89.1% reduction for four filters when measured with a quantum sensor (QS, Delta‐T).

All three plants used in one round were inside a radius of 1 m, and had a similar size and orientation. Small ventilators were placed close to the filters and switched on automatically by a datalogger (CR10, Campbell Scientific) as soon as the control plant outside the filters showed differing mean leaf temperatures, measured as described earlier.

PPFD intensity at the time of the experiment was determined with a microclimate station that was approximately 25 m distant. The procedure was repeated six times (11–18 August 2012). Each time the neutral filters were left in place for 45 min, and afterwards, pieces of two different leaves per plant were GA‐fixed (as described) and analysed under a light microscope.

#### Effect of temperature/irradiation combinations on rCP

Potted plants were placed in a measurement cuvette (3010‐A, Walz, Effeltrich, Germany), which was mounted in a gas‐exchange fluorescence measurement system (GFS 3000) from the same manufacturer, and the leaf temperature was set to +5 °C (at 70% relative humidity and 350 ppm CO_2_ concentration). After the temperature remained stable for about 12 min, the cuvette was opened and a single leaf was harvested for fixation with GA (as described for the daytime experiment). Then the cuvette was closed and the temperature set to 25 °C. After the temperature reached and remained at the set value for another 12 min, again, the cuvette was opened and a single leaf was taken and fixed. The same plant was subjected to two more changes in temperature, 37 °C and 15 °C, including removal of leaves and fixation. The temperature adjustment was performed at a speed of 1 K·min^−1^. The entire procedure was conducted both in full darkness and at two different irradiation intensities (PPFD 900 and 1800 *μ*mol photons·m^−2^·s^−1^) provided by the light‐emitting diode (LED) array/PAM‐Fluorometer (3055‐FL, Walz) that was fixed to the cuvette. In darkness and with each illumination level, the procedure was repeated three times.

### CPs and photosynthesis: effects of altered CO_2_/O_2_ ratio on rCP

The influence of the ambient gas composition on rCP was assessed again using the GFS 3000, which allows the adjustment of the CO_2_ concentration of the measurement gas for whole plants. Whole (potted) individuals were mounted in the measurement cuvette (3010‐A). Following a 15 min dark‐adaptation period, the potential efficiency of photosystem II (F_v_/F_m_) was automatically determined by the system to ensure that the selected individuals were healthy and not photoinhibited. Then, the irradiation was increased stepwise up to PPFD 1800 *μ*mol photons·m^−2^·s^−1^ resulting in the recording of a light–response curve. During the entire time period (approx. 50 min) the air temperature inside the measurement cuvette was set to 25 °C and the humidity to 10 000 ppm H_2_O. The procedure was conducted at a flow rate of 750 *μ*mol·s^−1^ and at four different CO_2_/O_2_ ratios (gas mixtures; Table 1), and was repeated three times for each gas mixture.

**Table 1:** CO_2_ and O_2_ concentration in the gas mixtures 1–4 applied to *R* *anunculus glacialis* individuals

Mix.	CO_2_ (ppm)	O_2_ (%)	Anticipated effect on key photosynthetic reactions
1	50	21	Photorespiration ↑
2	2000	21	Photorespiration ↓
3	2000	2	Photorespiration ↓↓, Mehler Pathway ↓
4	370	21	–

Gas mixture 3 (2000 ppm CO_2_/2% O_2_) was achieved with a gas cylinder (98% N_2_, 2% O_2_, 10 000 ppm CO_2_; Airliquide, Schwechat, Austria) that was connected to the GFS 3000. Following each treatment, a single leaf was GA‐fixed as described earlier. Four of the samples, each representing a different CO_2_/O_2_ ratio, were prepared for TEM analysis.

After the GA fixation, these samples were post‐fixed (1% OsO_4_), dehydrated in increasing ethanol concentrations, and embedded (Low Viscosity Embedding Kit, Serva, Heidelberg, Germany) as described by Holzinger *et al*. (2007a).

Ultrathin sections were post‐stained and examined with a TEM (Libra 120, Carl Zeiss, Oberkochen, Germany) at 80 kV. Images were captured with a CCD camera (Slow Scan, ProScan, Lagerlechfeld, Germany) and observed using iTEM 5.0 software (Olympus, Tokyo, Japan). The TEM analysis was aimed at observing CPs at the ultrastructural level, and no quantitative survey was conducted. The samples not prepared for TEM were analysed under light microscopy for their respective rCP values.

### Statistics

Pearson product–moment correlation coefficients (PCC), analysis of variance (anova) and the respective *post hoc* tests (Duncan test, Games–Howell test) were conducted using statistics software (SPSS 21, IBM, Armonk, NY, USA).

## Results

### Microclimate

**Figure 1:**
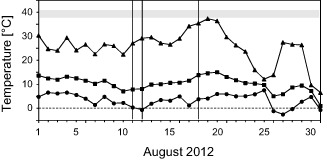
Leaf temperatures of *R* *anunculus glacialis* measured *in situ* (2563 m a.s.l.) during August 2012. For each day, the maximum (triangles), mean (squares) and minimum (circles) half‐hourly mean values are depicted. Vertical lines indicate the days on which the diurnal course of the occurrence of chloroplast protrusions was investigated. The horizontal grey band indicates the temperature threshold that caused initial impairment of photosynthetic functions (after Larcher & Wagner 1976; Larcher *et al*. 1997; Buchner *et al*. 2014).

From 1 to 23 August, the weather was stable, sunny and hot, with air and leaf temperatures rising each day at the investigation site. The maximum air and leaf temperatures recorded on 11, 12 and 13 August were about average for this month, whereas on 18 August it was particularly warm, leading to the third‐highest (36.4 °C) maximum leaf temperature measured during the entire month. Freezing temperatures were recorded during five nights (minimum: −3.4 °C). The highest half‐hourly mean leaf temperature (HHM) recorded on site during August 2012 was 37.3 °C (22 August; Fig. 1). The highest absolute leaf temperature measured in this period was 38.4 °C (19 August).

### Microscopic analyses

**Figure 2:**
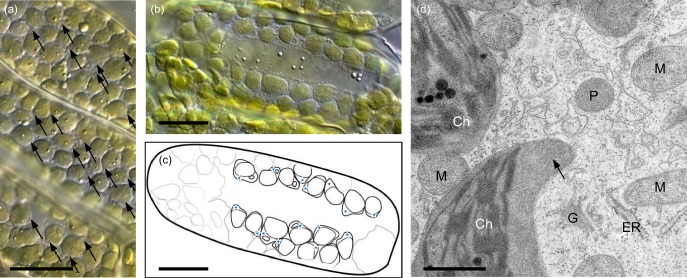
Microscopy images of chloroplast protrusions (CPs) in leaves of *R* *anunculus glacialis*. Whole individuals were irradiated (PPFD 1600 *μ*mol photons·m^−2^·s^−1^) and simultaneously exposed to different gas mixtures, characterized by certain CO_2_/O_2_ ratios. (a) light microscopy image (DIC illumination) of palisade cells treated with gas mixture 1 (50 ppm CO_2_/21% O_2_). Arrows indicate CPs, horizontal bar: 10 *μ*m. (b) DIC image of a single palisade parenchyma cell treated with gas mixture 4 (370 ppm CO_2_/21% O_2_). horizontal bar: 10 *μ*m. (c) schematic drawing of the same cell, blue stars indicate CPs. (d) transmission electron microscopy (TEM) image of a single palisade parenchyma cell (gas mixture 3: 2000 ppm CO_2_/2% O_2_). Ch, chloroplast; ER, rough endoplasmic reticulum; G, golgi stacks; M, mitochondria; P, peroxisome; pl, plastoglobuli; photosynthetically active photon flux density. arrow, chloroplast protrusion; horizontal bar: 1 *μ*m.

The observed CPs were beak‐like or lobe‐shaped and in many cases had invaginations (Fig. 2). No structures resembling the tubular, thin appearance of stromules (e.g. Köhler & Hanson 2000; Holzinger *et al*. 2007a) were observed.

TEM examination of the two gas treatment samples that showed the highest (gas mixture 4) and lowest (gas mixture 3) mean rCP in the light microscopy analysis, showed no signs of artefacts or damage because of the chemical fixation. Although rarer, CPs of variable shape and size were found not only in the gas mixture 4, but also in the gas mixture 3 samples (Fig. 2d). Cells in both samples contained large numbers of mitochondria.

No common difference in the appearance of CPs between the two samples was discernible.

### Diurnal courses of CP occurrence

**Figure 3:**
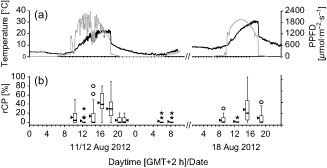
Effect of sampling time on rCP in *R* *anunculus glacialis*. (a) Diurnal courses of leaf temperature (black line) and irradiation (grey line) at the *in situ* experimentation site (2563 m a.s.l.) on 11–12 and 18 Aug 2012. (b) rCP values of the samples taken at the respective time. Black bars inside boxes represent medians; bottoms of boxes indicate the 25th, the tops the 75th percentile; whiskers extend to 1.5 times box‐height; circles stand for outliers, asterisks for extreme outliers that have values more than three times box‐height; triangles indicate arithmetic means.

The rCP of the samples taken from untreated plants *in situ* varied significantly with the time of day (Fig. 3a,b). The samples taken during the afternoon showed significantly (*P* < 0.05) increased rCP values (11 Aug, 16:26: rCP = 42.9%; 18 Aug, 15:14: rCP = 28.6%). These maximum values where measured in samples that experienced a leaf temperature averaged over 10 min (TL10) of 22 °C on the 11 Aug and a TL10 of 27.3 °C on 18 Aug.

rCP was lowest in the morning hours before sunrise, in the late evening, and during the night. Further, a distinct midday‐drop of rCP was detectable on both days (11 Aug, 12:18: rCP = 2.8%; 18 Aug, 12:42: rCP = 2.0%). The climate at the respective sampling times was characterized by high irradiation intensity and still rather low leaf temperature (11 Aug: TL10 = 12 °C/PPFD = 1685 *μ*mol photons·m^−2^·s^−1^; 18 Aug: TL10 = 20 °C/PPFD = 1884 *μ*mol photons·m^−2^·s^−1^).

During the afternoon, rCP values of single cells higher than 50% were measured frequently. The highest rCP value in a single leaf was measured in a sample taken at 15:14 on 18 Aug (47.7%). The lowest mean rCP value (1.4%) was measured on 12 Aug before sunrise (08:15). A summary of the individual rCP values including statistics is given as Supporting Information (Table S1).

### Effect of leaf temperature and irradiation intensity on rCP

**Figure 4:**
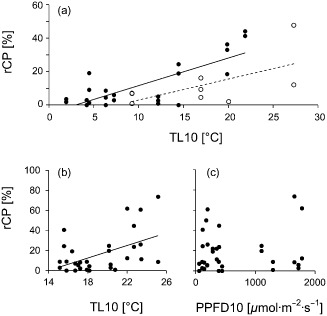
Chloroplast protrusions in leaves of *R* *anunculus glacialis* in response to leaf temperature and irradiation intensity. rCP values were taken (a) during the daytime and (b, c) during the *in situ* shading experiments. (a) Correlation diagram between mean rCP and the 10 min mean leaf temperature (TL10). Solid symbols, solid line: 11–12 August, *R* ^2^ = 0.6285, *n* = 25. Open symbols, dashed line: 18 August, *R* ^2^ = 0.3866, *n* = 9. (b) Correlation diagram between rCP and TL10 (R ^2^ = 0.2424, *n* = 32) and (c) between rCP and the 10 min mean photosynthetically active photon flux density (PPFD10) (*R* ^2^ = 0.0044, *n* = 32). rCP values represent leaf sample means.

The microclimate data for the samples taken *in situ* on 11–12 Aug indicate that rCP and the 10 min leaf‐temperature mean (TL10) correlated strongly (Fig. 4a). The PCC between rCP and TL10 (0.793; *P* < 0.01; *n* = 25) was almost twice as high as between rCP and the 10 min irradiation‐intensity mean (PPFD10) (0.405; *P* = 0.44; *n* = 25), which did not correlate significantly. On 18 Aug 2012 similar results were obtained (Figs 3a,b and 4a). Although the PCC between rCP and TL10 (0.632; *P* = 0.73; *n* = 9) was lower than for the data from 11–12 Aug, the correlation was again markedly higher than between rCP and PPFD10 (0.450; *P* = 0.224; *n* = 9). However, because of the relatively small number of samples for this day, the calculated correlations were not statistically significant.

### Effect of *in situ* shading on rCP

*In situ* shading of plants clearly showed the divergent influences of irradiation intensity and temperature on rCP. Comparison of the individual TL10 values showed that the difference between the shaded plants and the control plants reached up to 4 K in the same time period. The leaf temperatures underneath two and four neutral filters varied only by 1.7 K at the maximum. On average, the TL of all the plants was 19.5 °C during the 10 min and 19.4 °C during the 30 min period before the sampling. The reference irradiation intensity measured immediately before sampling at the nearby microclimate station (PPFD10) was between 1102 and 2020 *μ*mol photons·m^−2^·s^−1^.

The rCP values, obtained during the microscopic assessment of the samples from the shading experiment, correlated to some extent with the TL10 values of the respective samples (Fig. 4b). Consistent with the results of the experiment on the effect of daytime on rCP, the PPFD10 value again appeared to have a much lower, if not negligible influence on rCP (Fig. 4c).

Calculating the PCC between rCP and TL10 showed that the correlation, although lower than in the experiment on the effect of time of day on rCP, was highly significant (0.491; *P* < 0.01, *n* = 32). The PCC between rCP and PPFD10 on the other hand was very low and not significant (0.066; *P* = 0.718, *n* = 32). Unlike in the other experiments, the values of the 20 min TL (TL20) correlated slightly better with rCP than the 10 min TL (0.512; *P* < 0.01, *n* = 32).

### Effect of temperature/irradiation combinations on rCP

**Figure 5:**
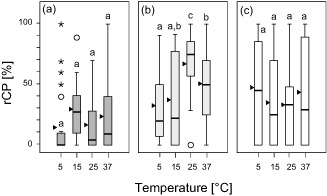
Chloroplast protrusions in response to leaf temperature and irradiation intensity. Boxplots showing rCP during stepwise‐increased leaf temperatures. The experiments were conducted (a) in darkness (dark grey), (b) at PPFD 900 (light grey), and (c) at PPFD 1800 *μ*mol photons·m^−2^·s^−1^ (white boxes). For each irradiation level, significant differences between mean values (anova, Duncan test, *P* < 0.05) are indicated by different characters; triangles indicate arithmetic means.

The light microscopy analysis of the 35 leaf samples obtained in the temperature/irradiation combinations experiment showed that both factors led to strong differences in rCP, as can be seen in Fig. 5. The four temperature steps as well as the continuous approximation towards the plateau phases, after which the samples were taken, significantly affected the number of CPs found in the respective samples.

The samples that underwent the temperature course at PPFD 900 and 1800 *μ*mol photons·m^−2^·s^−1^ showed a significantly (*P* < 0.05) higher rCP, with a maximum of 91.4%, than those taken in darkness. In contrast, the doubling of the PPFD 900 *μ*mol photons·m^−2^·s^−1^ led to a distinct decrease in mean rCP at 25 and 37 °C (Fig. 5c).

Furthermore, Fig. 5 shows that 25 °C combined with intermediate irradiation intensity (PPFD 900 *μ*mol photons·m^−2^·s^−1^; Fig. 5b) led to the highest mean rCP (66.6%, *n* = 28).

The lowest mean rCP was measured in the three leaf samples taken in darkness at the 5 °C temperature step (14.0%, *n* = 30).

None of the plants treated showed any macroscopic signs of damage or stress after having endured the large temperature changes to which they were exposed in this experiment. A summary of the individual rCP values as determined during the experiment is given as Supporting Information Table S2.

### CPs and photosynthesis; Effects of altered CO_2_/O_2_ ratio on rCP

**Figure 6:**
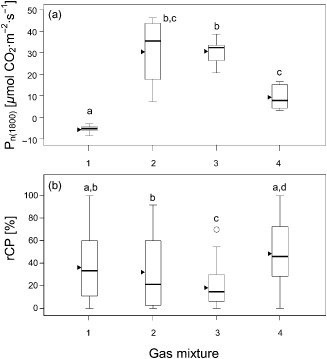
Chloroplast protrusions and net photosynthesis rate in response to the ambient air CO_2_/O_2_ ratio. Data were derived from light–response curves that were obtained at different CO_2_/O_2_ ratios of the ambient air: Mixture 1: 50 ppm CO_2_/21% O_2_; Mixture 2: 2000 ppm CO_2_/21% O_2_; Mixture 3: 2000 ppm CO_2_/2% O_2_; Mixture 4 (control): 370 ppm CO_2_/21% O_2_. PPFD was increased stepwise from 0 to 1800 *μ*mol photons·m^−2^·s^−1^. (a) Net photosynthesis rate P_n(1800)_ at PPFD 1800 *μ*mol photons·m^−2^·s^−1^ in response to the different CO_2_/O_2_ ratios. (b) rCP as determined at the end of the light–response curve as a function of the different CO_2_/O_2_ ratios). Significant differences between mean values (anova, Games–Howell *post hoc* test, *P* < 0.05) are indicated by different letters; triangles indicate arithmetic means.

The mean net assimilation rate P_n(1800)_ in the four different gas mixtures matched the availability of CO_2_ inside the measurement cuvette (Fig. 6a,b). Under normal conditions (gas mixture 4) the mean P_n(1800)_ was 9.4 *μ*mol CO_2_·m^−2^·s^−1^. When CO_2_ concentration was increased to 2000 ppm, the mean P_n(1800)_ also significantly increased to 30.5 and 30.8 *μ*mol CO_2_·m^−2^·s^−1^ in gas mixtures 2 and 3, respectively. P_n(1800)_ was negative (−5.7 *μ*mol CO_2_·m^−2^·s^−1^) at 50 ppm CO_2_ (gas mixture 1).

The microscopy analysis of the leaf samples taken from the individual plants showed that rCP differed significantly between some of the groups in response to the gas treatment (Fig. 6a,b). Reducing the CO_2_ level to 50 ppm (gas mixture 1, Fig. 2) led to an rCP of 36.7%, similar to the result obtained with gas mixture 3 (2000 ppm CO_2_/21% O_2_), which led to a mean rCP of 32.4%.

Whereas palisade cells containing no CPs were observed in all four groups, only the plants treated with an immensely increased CO_2_/O_2_ ratio (gas mixture 3) yielded no photographed single cell containing more than 70% rCP, and also the lowest mean rCP (18.7%). The treatment with gas mixture 4 (representing normal conditions) led to the highest mean rCP of 48.9%. This group also included the leaf sample showing the highest abundance of CPs of all leaf samples that were visually analysed (91.9%; Fig. 2b,c, Supporting Information Table S3).

## Discussion

### Microclimate

Although August 2012 was one of the warmest months ever recorded in Austria (ZAMG 2012), leaf temperature thresholds known to prevent net CO_2_ uptake in *R. glacialis* (38–40 °C; Larcher & Wagner 1976; Larcher *et al*. 1997) were recorded only once during the entire investigation period. However, leaf temperatures exceeding the optimum temperature range of P_n_ in *R. glacialis* (15–25 °C at PPFD > 555 *μ*mol photons·m^−2^·s^−1^; Moser *et al*. 1977; Larcher *et al*. 1997) were measured frequently. The measured minimum leaf temperature (−3.5 °C) lies well above the temperature at which summer frost leads to incipient damage in *R. glacialis* (−7.9 °C; Taschler & Neuner 2004).

### Diurnal course of CP formation

The very low rCP values in the midday samples (Fig. 3b) might be explained by the combination of high PPFD intensities with low TL. These are environmental conditions under which cold‐induced photoinhibition might occur in leaves of *R. glacialis*. Streb *et al*. (2005) showed that at TL 10 °C the maximum P_n_ is reached at PPFD 700 *μ*mol photons·m^−2^·s^−1^ followed by a steady decline of P_n_ if the irradiation intensity is increased further. From Fig. 3 it can be seen that TL was as low as 10 °C just half an hour before the midday sampling (11 August) and PPFD had already reached approximately 2000 *μ*mol photons·m^−2^·s^−1^ at this time. During 18 August, a TL of 15 °C, that is a temperature that leads to an increased P_n_ even at PPFD 2000 *μ*mol photons·m^−2^·s^−1^ (Streb *et al*. 2005), was reached more than 1 h before the midday sampling. Still, of the four samplings conducted on that day, these samples showed the highest PPFD values in relation to the leaf temperature, and it showed the same decline in rCP as did the midday samples of the first session.

The temperature‐related increase in rCP appears to have shifted slightly towards higher temperatures between the two sessions (Fig. 3). The increase in TL within the week between the two sessions might have already led to physiological modulation, resulting in the observed shift.

The temperatures that led to the highest rCP values match the optimum temperature range of P_n_ determined for *R. glacialis* leaves (Moser *et al*. 1977). However, other biochemical processes taking place inside the chloroplast or the surrounding cell might likewise benefit from these temperatures.

In summary, under field conditions, CP formation showed a pronounced diurnal pattern and was clearly temperature‐dependent. Moderate leaf temperatures in the range of the photosynthetic temperature optimum promoted CP formation. Although CPs could also be observed to a small extent in darkness, the formation of significant rCP values required a certain level of irradiation. Under high irradiation conditions combined with low temperature, rCP was significantly reduced, indicating a possible role of photosynthetic functions in the formation of CPs.

### Effect of leaf temperature and irradiation intensity on rCP

#### Effect of *in situ* shading on rCP

Shading of whole individuals clearly resulted in no linear correlation between rCP and PPFD. The mean rCP above 40% measured in samples that experienced less than 400 *μ*mol photons·m^−2^·s^−1^ while having ideal TL for photosynthesis (Moser *et al*. 1977) again suggests that CPs in *R. glacialis* occur mainly in moderate temperature and irradiation conditions.

The lower correlation between TL10 and rCP (Fig. 4b) compared with the results for the diurnal course (Fig. 4a) could be explained by the lower TL range experienced by the plants in the shading experiment.

#### Effect of temperature/irradiation combinations on rCP

The results from the temperature/irradiation combinations experiment confirm the findings of the *in situ* experiments (day course and shading experiments), that leaf temperature significantly influences the formation of CPs, with temperatures around 25 °C and moderate irradiation most likely to promote CPs (Fig. 5).

Earlier experiments where small cross sections of *R. glacialis* leaf tissue were temperature‐treated using a specially designed temperature‐controlled object slide have shown that in darkness a TL above 25 °C resulted in a rCP decrease (Buchner *et al*. 2007a, 2007b). At moderate irradiation, this phenomenon was likewise observed in the present experiment. However, it has been shown that this decline in rCP above 25 °C is not a general peculiarity of CPs. In *A. thaliana*, temperatures of up to 45 °C may still lead to an increase in rCP (Holzinger *et al*. 2007a).

High irradiation intensity led to fewer CPs at 25 and 37 °C than did moderate intensity (Fig. 5). While the resulting difference in rCP was not significant for this experiment, earlier experiments conducted with PPFD 500 and 2000 *μ*mol photons·m^−2^·s^−1^ *in vitro* led to a statistically significant reduction in rCP at the higher irradiation intensity (Buchner *et al*. 2007a). The change in rCP with the respective temperature appears inconclusive in the high‐irradiation samples. However, it reinforces the assumption that high irradiation intensity may have a suppressing influence on CP formation.

While some of the results of this experiment are not statistically significant, their consistency with the *in situ* as well as the *in vitro* results (Buchner *et al*. 2007a, 2007b; Holzinger *et al*. 2007a) are highly confirmative.

### CPs and photosynthesis; effect of altered CO_2_/O_2_ ratio on rCP

#### CPs and carbon assimilation

It is obvious that CP formation is in some way linked to photosynthesis, although not necessarily exclusively. However, the underlying mechanisms remain unclear. The formation of CPs was almost indirectly proportional to P_n_, which increased threefold when the CO_2_ concentration was high (Fig. 6). However, with respect to the decrease in rCP at high irradiation (see earlier), the photosynthetic assimilation rate alone cannot explain the changes in rCP.

As demonstrated, CP formation is highest under moderate and ‘normal’ ambient conditions with regard to leaf temperature, irradiation intensity and the CO_2_/O_2_ ratio. These conditions favour high physiological activity, which is characterized by high turnover and transport of metabolites. The formation of CPs increases both the chloroplast envelope surface and the stroma volume, and may therefore support high physiological activity and transport of metabolites from the chloroplast to the cytoplasm and *vice versa*. As in darkness, the metabolic exchange through the chloroplast envelope does not completely stagnate, it is conceivable that CP formation in darkness may be connected to the export of photosynthetic products, for example hexoses from the chloroplast (Schleucher *et al*. 1998) during the degradation of transitory starch, and related transport processes.

#### CPs and photorespiration

Ribulose‐1,5‐bisphosphate (RuBP) carboxylase/‐oxygenase (rubisco) is a bifunctional enzyme that catalyses not only the reduction of RuBP, but also its oxygenation, thereby producing 2‐phosphoglycolate, which is dephosphorylated in the chloroplast, and the resulting glycolate is transported into peroxisomes for further processing in the photorespiratory pathway (see Mhamdi *et al*. 2012). This pathway requires the cooperation of three cell organelles (chloroplast, peroxisome and mitochondrion), which are often found in close spatial proximity, as observed for *R. glacialis* (Lütz 1987) and *Deschampsia antarctica* (Gielwanowska & Szczuka 2005). The possibility that this pathway benefits and hence induces close proximity of the chloroplast membrane to mitochondria and peroxisomes via CPs or stromules has been suggested in several publications (Buchner *et al*. 2007a; Holzinger *et al*. 2007a; Sage & Sage 2009; Lütz 2010; Hanson & Sattarzadeh 2011; Lütz *et al*. 2012).

Gas mixture 3 (2000 ppm CO_2_/2% O_2_), which contained a 56.8 times higher CO_2_/O_2_ ratio than the standard gas mixture 4 (370 ppm CO_2_/21% O_2_) is known to induce a drastic reduction of both photorespiration and the Mehler reaction (Makino 2002), where electrons are transferred to oxygen, resulting in the formation of the superoxide radical O_2_⋅^−^. The correlation of the low rCP with the presumably low photorespiration rates and low activity of the Mehler reaction caused by gas mix 3 indirectly supports a possible connection between CP formation and these pathways.

On the other hand, gas mixture 1 (50 ppm CO_2_/21% O_2_), aimed at strongly increasing oxygenation, led to a slightly lower mean rCP than the standard CO_2_/O_2_ ratio (370 ppm CO_2_/21% O_2_). Furthermore, the aforementioned midday drops in rCP, as observed during the day course experiment, took place under conditions with the potential to increase photorespiratory activity. However, the difference in rCP between the treatment with gas mixture 1, which led to a negative P_n_ and the treatment with the standard gas mixture, which led to a distinctly positive P_n_ is minimal. If photorespiration was enhanced by gas mixture 1 and CP formation is promoted by photorespiration, rCP should have increased significantly in comparison with gas mixture 4, which represented normal conditions. That was, however, not the case. Therefore, from our results, a key role of the photorespiratory pathway in the formation of CPs cannot be inferred.

The enzyme catalase plays a decisive role in the photorespiratory pathway (Lütz 1987; Willekens 1997), and is located in peroxisomes, which frequently occur close to chloroplasts in *R. glacialis* (Lütz 1987). Therefore, for future experiments concerning CPs and their possible role in photorespiration, it will be illuminating to determine catalase activity and relate the results to CP formation.

## Conclusion

For the first time, it has been shown that CPs (1) follow a pronounced diurnal rhythm under fully natural conditions *in situ*. Furthermore, the results indicate (2) that the occurrence of CPs is directly linked to leaf temperature. The immediate influence of irradiation on rCP seems negligible. However, taking the *ex situ* results into account, a certain level of irradiation might be required prior to or during changes in temperature, in order to show its direct influence on rCP. (3) While the data presented here suggest that CP formation is not directly linked to P_n_, some oxygenation process appears to favour their formation. The involvement of photorespiration remains, however, ambiguous.

Our results provide evidence that under field conditions, moderate temperature and irradiation levels appear to promote high rCP and that CP formation is related neither to temperature nor to irradiation stress in *R. glacialis*.

## Supporting information


**Table S1.** Effect of day time on the formation of chloroplast protrusions in leaf mesophyll cells of *R. glacialis*. Study site: Timmelsjoch (2563 m a.s.l., Ötztal Alps, Tirol, Austria). Data originate from 11/12 August and 18 August 2012. rCP: mean value of the relative proportion of chloroplasts with at least one chloroplast protrusion at the respective sampling time. For each day course significant differences between groups based on One‐way anova and Duncan's *post hoc* test (*P* < 0.05) are indicated by different letters.
**Table S2.** Effect of leaf temperature and irradiation intensity (PPFD) on the formation of chloroplast protrusions in leaf mesophyll cells of *R. glacialis*. Potted plants were exposed to a series of different temperatures in darkness and at different irradiation intensities (PPFD = 900 and 1800 *μ*mol photons·m^−2^·s^−1^) and mean rCP, the relative proportion of chloroplasts with at least one chloroplast protrusion, was determined. *n*, sample size. For each irradiation intensity, significant (*P* < 0.05) differences between rCP values originating from different temperature levels are indicated by different letters (One‐way anova followed by Duncan's *post hoc* test).
**Table S3.** Effect of CO_2_/O_2_ ratio on net photosynthesis rate and the formation of chloroplast protrusions in leaf mesophyll cells of *R. glacialis*. Potted plants were exposed to different gas mixtures while light–response curves were conducted. The gas mixtures were characterized by different CO_2_/O_2_ ratios. Immediately after the end of the experiments (at PPFD 1800 *μ*mol photons·m^−2^·s^−1^), mean rCP, the relative proportion of chloroplasts with at least one chloroplast protrusion, was determined. *n*, sample size; P_n(1800)_ net assimilation rate at PPFD 1800 *μ*mol photons·m^−2^·s^−1^. Significant (*P* < 0.05) differences between rCP and P_n(1800)_ values originating from different gas mixtures are indicated by different letters (one‐way anova followed by Duncan's *post hoc* test).
