# Supplementary material for: Chloroplast protrusions in leaves of R anunculus glacialis L. respond significantly to different ambient conditions, but are not related to temperature stress
Source: Plant Cell Environ. 2015 Jan 23;38(7):1347–56. doi: 10.1111/pce.12483 (PMC5098225; doi:10.1111/pce.12483)
Supplement: Supplementary file 1 — Table S1. Effect of day time on the formation of chloroplast protrusions in leaf mesophyll cells of R. glacialis. Study site: Timmelsjoch (2563 m a.s.l., Ötztal Alps, Tirol, Austria). Data originate from 11/12 August and 18 August 2012. rCP: mean value of the relative proportion of chloroplasts with at least one chloroplast protrusion at the respective sampling time. For each day course significant differences between groups based on One‐way anova and Duncan's post hoc test (P < 0.05) are indicated by different letters. Table S2. Effect of leaf temperature and irradiation intensity (PPFD) on the formation of chloroplast protrusions in leaf mesophyll cells of R. glacialis. Potted plants were exposed to a series of different temperatures in darkness and at different irradiation intensities (PPFD = 900 and 1800 μmol photons·m−2·s−1) and mean rCP, the relative proportion of chloroplasts with at least one chloroplast protrusion, was determined. n, sample size. For each irradiation intensity, significant (P < 0.05) differences between rCP values originating from different temperature levels are indicated by different letters (One‐way anova followed by Duncan's post hoc test). Table S3. Effect of CO2/O2 ratio on net photosynthesis rate and the formation of chloroplast protrusions in leaf mesophyll cells of R. glacialis. Potted plants were exposed to different gas mixtures while light–response curves were conducted. The gas mixtures were characterized by different CO2/O2 ratios. Immediately after the end of the experiments (at PPFD 1800 μmol photons·m−2·s−1), mean rCP, the relative proportion of chloroplasts with at least one chloroplast protrusion, was determined. n, sample size; Pn(1800) net assimilation rate at PPFD 1800 μmol photons·m−2·s−1. Significant (P < 0.05) differences between rCP and Pn(1800) values originating from different gas mixtures are indicated by different letters (one‐way anova followed by Duncan's post hoc test). [file PCE-38-1347-s001.zip › PCE_12483_Suppl 2_final.pdf]

Supplement 2. Effect of leaf temperature and irradiation intensity (PPFD) on the formation of chloroplast protrusions in leaf mesophyll cells of *R. glacialis*. Potted plants were exposed to a series of different temperatures in darkness and at different irradiation intensities (PPFD = 900 and 1800 µmol photons·m^{-2}·s^{-1}) and mean rCP, the relative proportion of chloroplasts with at least one chloroplast protrusion, was determined. SE standard error; n sample size. For each irradiation intensity significant ($P < 0.05$) differences between rCP values originating from different temperature levels are indicated by different letters (One-way ANOVA followed by Duncan's Post-hoc test).

PPFD = 0 µmol photons·m^{-2}·s^{-1}				
T [°C]	5	15	25	37
rCP [%]	14.0	28.9	16.1	23.0
SE	4.5	5.1	4.0	5.7
n	31	20	32	30
Sign. (P<0.05)	a	a	a	a

PPFD = 900 µmol photons·m^{-2}·s^{-1}				
T [°C]	5	15	25	37
rCP [%]	31.6	36.5	66.6	49.8
SE	6.0	6.6	4.9	5.6
n	26	32	29	29
Sign. (P<0.05)	a	a,b	c	b

PPFD = 1800 µmol photons·m^{-2}·s^{-1}				
T [°C]	5	15	25	37
rCP [%]	47.0	34.0	32.3	43.0
SE	7.1	6.2	5.4	7.4
n	30	30	31	32
Sign. (P<0.05)	a	a	a	a
